# A Existência Prévia de Doenças do Aparelho Circulatório Acelera a Mortalidade por COVID-19?

**DOI:** 10.36660/abc.20200486

**Published:** 2020-07-28

**Authors:** Carlos Dornels Freire de Souza, Thiago Cavalcanti Leal, Lucas Gomes Santos

**Affiliations:** 1 Universidade Federal de Alagoas ArapiracaAL Brasil Universidade Federal de Alagoas - Campus Arapiraca, Arapiraca, AL - Brasil

**Keywords:** Coronavirus, COVID-19, Pandemia, Síndrome Respiratória Aguda Grave/complicações, Comorbidades, Fatores de Risco, Diabetes, Hipertensão, Dispnea

**Caro Editor,**

Os primeiros casos de doença de coronavírus 2019 (COVID-19) foram identificados na metrópole de Wuhan, capital da província de Hubei, na República Popular da China.^1^ Nela, observou-se um surto de pneumonia de rápida progressão e de origem indeterminada associada à exposição comum ao mercado de frutos do mar da cidade.^1^ Em 31 de dezembro de 2019, a China notificou o surto à Organização Mundial da Saúde (OMS).^1^ Um mês depois, em 30 de Janeiro, a OMS declarou situação de emergência internacional e, em 11 de março, foi declarada pandemia pela doença.^2^

No Brasil, o primeiro caso foi confirmado em 26 de fevereiro de 2020, em São Paulo. Em 17 de março, foi registrada a primeira morte no país e, três dias depois (20 de março), o Ministério da Saúde reconheceu a transmissão comunitária em todo território nacional. Em 15 de maio, o Brasil ocupava a sexta posição mundial em casos acumulados, com mais de 200.000 infectados e mais de 13.000 mortes.^3^

Dentre os aspectos mais relevantes a serem observados no curso da pandemia são os grupos de maior risco, dos quais se destacam os indivíduos com a idade superior a 60 anos e aqueles com comorbidades cardiovasculares como fatores de pior prognóstico e maior letalidade quando infectados pelo novo coronavírus.^4^

Este estudo objetivou analisar a associação entre a existência prévia de doenças do aparelho circulatório e o tempo (em dias) entre o início dos primeiros sintomas e a data do óbito por COVID-19.

Trata-se de um estudo de caso-controle envolvendo dados de 374 óbitos por COVID-19 registrados no estado de Pernambuco. Os dados foram obtidos da página eletrônica de monitoramento da COVID-19 do estado (https://dados.seplag.pe.gov.br/apps/corona.html), em 07 de maio de 2020. Após a coleta, o banco de dados passou por ajustes das variáveis, que consistiu na avaliação dos sinais/sintomas e comorbidades. Após a adequação, 197 indivíduos possuíam doença do aparelho circulatório prévia, dos quais 187 apresentavam data do início dos sintomas e data do óbito. Esses indivíduos compuseram o grupo de casos. Para a composição do grupo controle, foram selecionados 187 óbitos que não possuíam comorbidades relatadas. A seleção desses óbitos foi aleatória, obedecendo a data de início dos primeiros sintomas.

No estudo, foram consideradas as seguintes variáveis: comorbidades existentes (nenhuma, uma, duas e três ou mais) e o tempo (em dias) entre a data dos primeiros sintomas e o óbito por COVID-19. Para a análise estatística, foi utilizado o teste de Kolmogorov-Smirnov para a avaliação inicial da normalidade dos dados. Uma vez constatada a violação do pressuposto de distribuição gaussiana, a associação entre as variáveis foi avaliada pelo teste não paramétrico U de Mann-Whitney. As análises consideraram significância de 5% e foram realizadas com o auxílio do software SPSS versão 24.0 (IBM Corporation). Por utilizar dados de domínio público, nos quais não é possível a identificação dos indivíduos, este estudo dispensou a aprovação pelo Comitê de Ética em Pesquisa.

**Figura 1:**
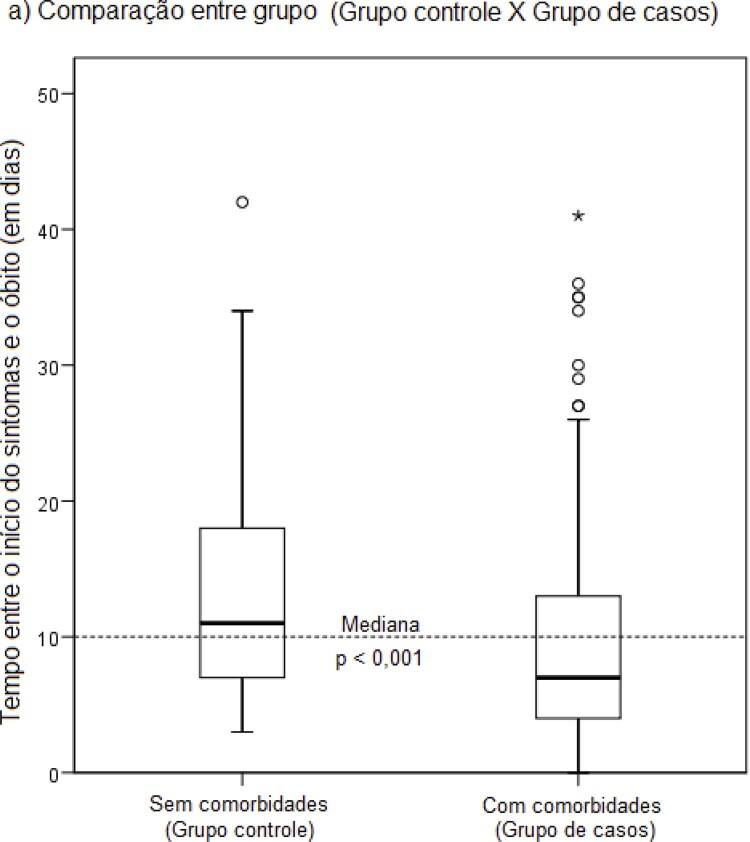
– *Comparação entre o número de dias entre o início dos primeiros sintomas e o óbito por COVID-19, segundo presença/ausência de comorbidades. Brasil, 2020.*

A média e desvio-padrão (média ± DP) e mediana e intervalo interquartil (mediana – IIQ) de dias entre o início dos primeiros sintomas e a data do óbito de toda a população do estudo (n = 374) foram 11,52 (± 7,75) e 10 (IIQ 10), respectivamente. Do grupo casos, 38 (20,3%) possuíam apenas uma doença do aparelho circulatório; 79 (42,2%) possuíam duas comorbidades/fatores de risco e 70 (37,5%) possuíam três ou mais comorbidades/fatores de risco. Salienta-se que pelo menos uma das comorbidades estava relacionada com o sistema circulatório ( Figura 1 ).

Observou-se diferença significativa entre o número de dias entre o início dos primeiros sintomas e o óbito ao comparar os dois grupos. Os valores observados no grupo controle (média ± DP= 13,32 ± 7,2; mediana – IIQ= 11 – 11) foram superiores ao grupo que possuía comorbidades relatadas (média ± DP = 9,73 ± 7,8; mediana – IIQ = 7 – 9) ( Figura 1 ).

O presente estudo aponta para uma progressão mais rápida da COVID-19 em quem possui comorbidades cardiovasculares, com uma média de dias do início dos primeiros sintomas ao óbito inferior em quase quatro dias (3,9 dias na média e 4,0 na mediana), quando comparados o grupo que possuía enfermidades cardiovasculares prévias e o grupo controle. Este processo decorre dos efeitos do SARS-CoV-2 no organismo humano, como a ligação do vírus à enzima conversora de angiotensina 2 (ECA2) encontrada nas superfícies das células cardíacas, renais e pulmonares.^4^

A exposição das glicoproteínas relacionadas ao novo coronavírus à ECA2 promove a sua internalização junto com o vírus, o que diminui a densidade de ECA2 na membrana^5 , 6^ e consequentemente o efeito cardioprotetor relacionado à hipertrofia cardíaca, à fibrose miocárdica e à inflamação. Nesse sentido, associa-se a redução de ECA2 à exacerbação das cardiopatias existentes, tais como insuficiência cardíaca e hipertensão arterial, contribuindo com a mais rápida progressão e o agravamento do quadro clínico respiratório e cardiovascular dos indivíduos com COVID-19.

Com base nos resultados observados, a presença de comorbidades cardiovasculares acelera a mortalidade por COVID-19. Ademias, outros estudos ainda devem ser realizados a fim de mensurar o impacto de cada doença cardiovascular no risco de mortalidade.
