# Supplementary material for: A Existência Prévia de Doenças do Aparelho Circulatório Acelera a Mortalidade por COVID-19?
Source: Arq Bras Cardiol. 2020 Jul 28;115(1):146–7. [Article in Portuguese] doi: 10.36660/abc.20200486 (PMC8384315; doi:10.36660/abc.20200486)
Supplement: Translation

# Translation: Does Existence of Prior Circulatory System Diseases Accelerate Mortality Due to COVID-19?

**Authors:** Carlos Dornels Freire de Souza, Thiago Cavalcanti Leal, Lucas Gomes Santos

**Affiliations:** 1 Brazil Universidade Federal de Alagoas - Campus Arapiraca, Arapiraca, AL - Brazil

**Keywords:** Coronavirus, COVID-19, Pandemics, Severe Acute Respiratory Syndrome/complications, Comorbidity, Risk Factors, Diabetes, Hypertension, Dispnea

**Dear Editor,**

The first cases of coronavirus disease 2019 (COVID-19) were identified in the metropolis of Wuhan, the capital of the province of Hubei, in the People’s Republic of China.^1^ An outbreak of rapidly progressive pneumonia of undetermined origin associated with common exposure to the city’s seafood market was observed.^1^ On December 31, 2019, China notified the World Health Organization (WHO) of the outbreak.^1^ One month later, on January 30, 2020, the WHO declared the situation an international emergency, and, on March 11, the disease was declared a pandemic.^2^

In Brazil, the first case was confirmed on February 26, 2020, in São Paulo. On March 17, the first death in the country was registered, and, three days later, on March 20, the Ministry of Health recognized community transmission throughout Brazilian territory. On May 15, Brazil held sixth place worldwide in total cases, with more than 200,000 individuals infected and more than 13,000 deaths.^3^

The most relevant aspects that should be observed during the course of the pandemic include the groups at greatest risk; in this group, individuals over the age of 60 and those with cardiovascular comorbidities stand out, as these factors are associated with worse prognosis and greater lethality when patients are infected with the novel coronavirus.^4^

The objective of this study was to analyze the association between the existence of previous circulatory system diseases and time (in days) from onset of first symptoms to date of death due to COVID-19.

This is a case-control study, involving data from 374 deaths due to COVID-19, registered in the state of Pernambuco, Brazil. Data were obtained from the state’s COVID-19 monitoring webpage (https://dados.seplag.pe.gov.br/apps/corona.html), on May 7, 2020. After collection, the database underwent adjustment of variables, which consisted of evaluation of signs/symptoms and comorbidities. Following adaptation, 197 individuals had prior circulatory system disease, 187 of which included date of onset of symptoms and date of death. These individuals constituted the case group. To form the control group, 187 deaths without related comorbidities were selected. Selection of these deaths was random, following the date of onset of symptoms.

In this study, the following variables were considered: existing comorbidities (none, one, two, and three or more) and time (in days) from onset of symptoms to death due to COVID-19. For statistical analysis, the Kolmogorov-Smirnov test was used for initial evaluation of data normality. When violation of the presupposed Gaussian distribution was found, association between variables was evaluated by nonparametric Mann-Whitney U test. Analyses considered a significance level of 5%, and they were carried out with the help of SPSS software, version 24.0 (IBM Corporation). Given that the study used public domain data, wherein it is not possible to identify the individuals, approval by the Research Ethics Committee was waived for this study.

Average and standard deviation (average ± SD) and median and interquartile range (median – IQR) of days from onset of first symptoms to date of death for the whole study population (n = 374) were 11.52 (± 7.75) and 10 (IIQ 10), respectively. In the case group, 38 (20.3%) had only one circulatory system disease; 79 (42.2%) had two comorbidities/risk factors, and 70 (37.5%) had three or more comorbidities/risk factors. It is worth highlighting that at least one of the comorbidities was related to the circulatory system ( Figure 1 ).

**Figure 1:** – *Comparison between number of days from onset of first symptoms to death due to COVID-19, according to presence or absence of comorbidities. Brazil, 2020.*

A significant difference was observed regarding the number of days from onset of first symptoms to death when comparing the two groups. The values observed in the control group (average ± SD = 13.32 ± 7.2; median – IQR = 11 – 11) were higher than those in the group with reported comorbidities (average ± SD = 9.73 ± 7.8; median – IQR = 7 – 9) ( Figure 1 ).

This study indicates more rapid progression of COVID-19 in patients with cardiovascular comorbidities; average number of days from onset of first symptoms to death was lower by almost four days (3.9 days for average and 4.0 days for median), when comparing the group that had prior cardiovascular disease to the control group. This process results from the effects of SARS-CoV-2 in the human body, such as the binding of the virus to angiotensin-converting enzyme 2 (ACE2) found in the surface of heart, kidney, and lung cells.^4^

Exposure of glycoproteins related to the novel coronavirus to ACE2 promotes internalization together with the virus, which diminishes the density of ACE2 in the membrane^5 , 6^ and, consequently, the cardioprotective effect related to cardiac hypertrophy, myocardial fibrosis, and inflammation. Accordingly, the reduction of ACE2 is associated with the exacerbation of existing heart diseases, such as heart failure and arterial hypertension, contributing to more rapid progression and worsening of respiratory and cardiovascular condition of individuals with COVID-19.

Based on the observed results, the presence of cardiovascular comorbidities accelerates mortality due to COVID-19. Furthermore, future studies need to be performed with the aim of measuring the impact of each cardiovascular disease on risk of mortality.
